# Free Spectacles for Children in Public Schools

**Published:** 1906-07-14

**Authors:** 


### Editor's Letter-Box.

[Our Correspondents are reminded that prolixity is a great bar to publication, and that brevity of style and conciseness of statement greatly facilitate early insertion.]

### FREE SPECTACLES FOR CHILDREN IN PUBLIC SCHOOLS.

Miss A. Susan Lawrence, Hon. Secretary to the Association for the Supply of Spectacles, etc., writes under date 4th inst. : I should be grateful if you would allow me to say a few words in answer to an article upon our society which appeared in last week's Hospital. The writer of the article tells us that he has nothing but approval of the scheme so far as the arrangements with the hospitals are concerned, and of the nomination in each district of an approved optician to supply prescribed glasses at proper prices. He thinks, however, that this part of the work would be better undertaken by the County Council. He is anxious that the parents themselves should pay for all glasses required. He points out the mischievous effects of indiscriminate charity, and asks under what guarantee or under what organisation we propose to administer our funds. Now, in the first place, the County Council have taken counsel's opinion on the extent of their powers under the Education Acts, and they have received a reply that the provision of spectacles is a matter entirely outside their province. They cannot, except at the risk of a surcharge, spend any money at all upon the supply of spectacles, and to make such an arrangement as outlined by your correspondent would cost a considerable sum in clerical work and in expenses of superintendence. It would no doubt be a good thing if, as the writer of the article suggests, further legislation could be obtained, but, until then, whatever is done, must be done by private persons and from private funds. Secondly, we think that the supply of spectacles in cases of real poverty is a legitimate form of charity. To supply glasses, or indeed any kind of medical appliance without careful and searching inquiry is, no doubt, wrong; but, in giving spectacles in cases of real need, we are only following in the steps of some of the most well-established and justly praised charities, such as the Surgical Aid Society, and many others. For the selection of cases, and for the distribution of help, we rely upon the school managers. Our plan is, that the chairman of managers, and he alone, can order what glasses may be required by the children in his school. He is responsible to the optician for the price of the glasses. The glasses are delivered at the school, and the managers can, if they choose, keep them back till paid for, or make what arrangements they think best for payment by instalments. The Association consider all applications from the managers for help, and whatever help we grant is paid to the managers to distribute as they think best. The managers are persons in touch with the children and accustomed to the administration of relief. We do not think that better advice than this could possibly be procured. We do not propose to undertake any work in schools where the managers are not willing to co-operate.

If this letter is not already too long, I should be glad to add one more word with regard to the prices at which we supply spectacles. We do not propose to supply the cheapest possible spectacles, or even the cheapest spectacles that we consider satisfactory. Our aim is to supply spectacles with frames and glasses exactly as prescribed by the various oculists, but at the cheapest possible rates. My committee had submitted to them a great number of spectacles, starting at 4d. a pair. They originally chose, under medical advice, frames of blue steel, by which spherical glasses could be supplied at 6d. a pair. When, however, we approached the hospitals, these frames did not meet with universal approval. Several specialists (among whom I must most gratefully mention Mr. Lang) were kind enough to spend a considerable time over the matter. In consequence, we now usually supply nickel frames for spherical glasses, and steel frames with curled ends for cylindrical glasses. This raises the price to 10d. and 2s. 6d. respectively. Some hospitals, however, have desired us to use the frames with curled ends for all cases. We, of course, comply, although this raises the price of simple spherical glasses to 1s. 6d. I have ventured to give these details because I feel that there is considerable force in your correspondent's remarks upon the wholesale price of glasses. I think, however, that we have now arranged for the cheapest glasses that it is possible to supply, consistent with working to prescription.

* * Miss Lawrence's letter abundantly displays the kindness of her own heart, but does not in the least degree remove our objections to her benevolent scheme. It rather increases them. If the State compel children to attend school, it becomes the duty of the State to see that the attendance is not rendered injurious to these children by lack of necessaries, and that any public provision of these necessaries, when they are not supplied by parents, is surrounded by proper restrictions and precautions against abuse. This is the principle of the Poor Law. The fact that the Legislature has so far neglected its plain duty to the children in order to use them as pawns in the game of party politics, is to be regretted; but will not be mended by the intervention of so-called "charity" for the diminution of evils for which Parliament is primarily responsible. To commit the selection of the cases in which spectacles may be "given" to the managers of the different schools, with no common or accepted principle to guide them, would be to inaugurate a reign of chaos; and the whole business is but one among many modern devices for the more complete demoralisation of already demoralised classes of the community.—Ed. T. H.

